# Gadolinium-Cyclic 1,4,7,10-Tetraazacyclododecane-1,4,7,10-Tetraacetic Acid-Click-Sulfonyl Fluoride for Probing Serine Protease Activity in Magnetic Resonance Imaging

**DOI:** 10.3390/molecules28083538

**Published:** 2023-04-17

**Authors:** Phuong Tu Huynh, Huy Duc Vu, Junghwa Ryu, Hee Su Kim, Hoesu Jung, Sung Won Youn

**Affiliations:** 1 Department of Radiology, Daegu Catholic University School of Medicine, 3056-6, Daemyung-4-Dong, Nam-gu, Daegu 42472, Republic of Korea; 2 Korea Basic Science Institute (Daegu Center), Kyungpook University, 80, Daehak-ro, Buk-gu, Daegu 41566, Republic of Korea; 3 Preclinical Research Center, Daegu-Gyeongbuk Medical Innovation Foundation (KMEDIhub), 88, Dongnae-ro, Dong-gu, Daegu 41061, Republic of Korea

**Keywords:** gadolinium contrast agent, elastase, serine protease, T1-weighted magnetic resonance imaging

## Abstract

Serine protease is linked to a wide range of diseases, prompting the development of robust, selective, and sensitive protease assays and sensing methods. However, the clinical needs for serine protease activity imaging have not yet been met, and the efficient in vivo detection and imaging of serine protease remain challenging. Here, we report the development of the gadolinium-cyclic 1,4,7,10-tetraazacyclododecane-1,4,7,10-tetraacetic acid-click-Sulfonyl Fluoride (Gd-DOTA-click-SF) MRI contrast agent targeting serine protease. The HR-FAB mass spectrum confirmed the successful formation of our designed chelate. The molar longitudinal relaxivity (r_1_) of the Gd-DOTA-click-SF probe (r_1_ = 6.82 mM^−1^ s^−1^) was significantly higher than that of Dotarem (r_1_ = 4.63 mM^−1^ s^−1^), in the range of 0.01–0.64 mM at 9.4 T. The in vitro cellular study and the transmetallation kinetics study showed that the safety and stability of this probe are comparable to those of conventional Dotarem. Ex vivo abdominal aortic aneurysm (AAA) MRI revealed that this probe has a contrast-agent-to-noise ratio (CNR) that is approximately 51 ± 23 times greater than that of Dotarem. This study of superior visualization of AAA suggests that it has the potential to detect elastase in vivo and supports the feasibility of probing serine protease activity in T1-weighted MRI.

## 1. Introduction

Serine proteases are one of the proteolytic enzyme families involved in a variety of physiological processes (including programmed cell death, cell differentiation, extracellular matrix (ECM) degradation, tissue remodeling, and inflammation) and cardiovascular diseases (including abdominal aortic aneurysm (AAA) and atherosclerosis) [1,2,3,4,5]. In the United States, AAA rupture, a potentially fatal vascular disease, causes approximately 15,000 deaths per year [6]. Elastase, one of the serine proteases, is not only used to induce AAA [7,8] but is also found at high concentrations in ruptured AAA specimens [9]. We believe that it could be used as a marker for AAA. Elastase is a representative single-chain polypeptide consisting of 240 amino acid residues and has a molecular weight of 25,900 Da; it contains four disulfide bridges and belongs to the serine protease family [10]. The importance of serine proteases and their close connection with disease have led to the development of robust, selective, and sensitive protease assays and sensor methods, including fluorescence-based techniques, colorimetric assays, electrochemical methods, enzyme-linked peptide protease assays, and liquid crystal (LC)-based protease assays [11]. None of them, however, are clinical imaging tools. It is still difficult to accurately identify and image serine proteases in living animals. The current techniques used to detect AAA are ultrasound, positron emission tomography (PET), computed tomography (CT), and magnetic resonance imaging (MRI) [6].

Magnetic resonance imaging (MRI) is a popular, non-invasive, and non-ionizing diagnostic imaging modality [12,13], and gadolinium (Gd) is used as the main contrast agent (CA) in MRI. The Gd complexes significantly enhance the longitudinal relaxation rate and then generate bright spots in T1-weighted MR images. The relaxivity and stability of Gd CAs are the first prerequisites for research on and development of new CAs. Among the common Food and Drug Administration (FDA)-approved MRI contrast agents in use are acyclic diethylenetriaminepentaacetic acid (Gd(III)-DTPA; Magnevist^®^; Bayer HealthCare Pharmaceuticals, Wayne, NJ, USA) and cyclic 1,4,7,10-tetraazacyclododecane-1,4,7,10-tetraacetic acid (Gd(III)-DOTA; Dotarem^®^; Guerbet, Aulnay-sous-Biois, France) [14,15]. Magnevist was reported to cause NSF, which was explained to be due to the separation of Gd ions from their chelating ligands [16,17,18,19,20,21]. Gd in free form is insoluble at physiological pH, leading to very slow systemic excretion, with the majority being deposited in bone, kidney, and liver, which can cause nephrotoxicity. In vivo, the transmetallation of the Gd complex occurs due to Gd^3+^ ions competing with various endogenous cations and endogenous anions for the ligand, which destabilizes the Gd complex and shifts the dissociation equilibrium toward its free components [14,22,23]. The high stability (logK = 25.3) of the DOTA backbone, thanks to eight-fold coordination and one water molecule binding directly to the central metal, and its kinetic inertness and dynamic stability are well known [24]. Thus, the DOTA backbone was chosen in our research because it is considered the “gold standard” ligand, with high stability in trivalent gadolinium chelation and the sulfonyl fluoride (SF) functional group, which specifically reacts with the hydroxy group (OH- group) of serine proteases such as elastase [25].

To target, isolate, and identify this specific protein or enzyme, activity-based probes (ABPs) are widely used. Sulfonyl fluoride (SF) has been the popular warhead for ABPs; it covalently binds not only to reactive serine but also to threonine, lysine, tyrosine, cysteine, and histidine residues in a context-specific manner. SF groups are popular residues to target and detect various active serine proteases. One of the commercially available SF reagents is (2-aminoethyl)benzene sulfonyl fluoride (AEBSF) [26,27,28,29]. 

We herein report the development of the novel Gadolinium-cyclic 1,4,7,10-tetraazacyclododecane-1,4,7,10-tetraacetic acid-click-sulfonyl fluoride (Gd-DOTA-click-SF)-based MRI contrast agent for sensing serine proteases, including elastase. This probe showed higher molar longitudinal relaxivity (r_1_) than Dotarem. The in vitro cell viability test demonstrated that there was no significant toxicity, with a viability of more than 80% at all concentrations, and the values observed for the probe were comparable to those of the clinical contrast agent Dotarem. The ability of the probe to react with elastase was studied with matrix-assisted laser desorption/ionization coupled to time-of-flight (MALDI-TOF) mass spectrometry. In the ex vivo abdominal aortic aneurysm (AAA) MRI study, the Gd-SF probe displayed higher signal intensity in T1-weighted MRI than conventional Dotarem, which demonstrates that this probe efficiently targets elastase to better visualize AAA models. 

## 2. Results

### 2.1. Synthesis of Gd-DOTA-Click-SF with Click Chemistry

#### 2.1.1. Procedure 1: Synthesis of DOTA-Click-SF with Click Chemistry 

In our study, we used the commercially available alkyne-functionalized sulfonyl fluoride activity-based probe (4-(2-(Hex-5-ynamido)ethyl)benzenesulfonyl fluoride) to achieve the click chemistry–based incorporation of 1,4,7,10-Tetraazacyclododecane-1,4,7-tris acetic acid-10-(azidopropyl-ethylacetamide) (azido-mono-amide-DOTA). 

At first, Cu^I^ was used as the catalyst for the click chemistry reaction between terminal alkyne and azide to form DOTA-click-SF, which was then allowed to react with GdCl_3_ to obtain the Gd-DOTA-click-SF complex. The product was then characterized using low-resolution FAB mass spectrometry. Cu-DOTA-click-SF mass calculated for C_33_H_48_FCuN_9_O_10_S [M]^+^: 844.25; found: 844.8, instead of the designed product (Figure S1). 

#### 2.1.2. Procedure 2: Synthesis of Gd-DOTA-Click-SF Probe Using Gd-Azido-DOTA and SF-Alkyne with Click Chemistry

**Figure 1:**
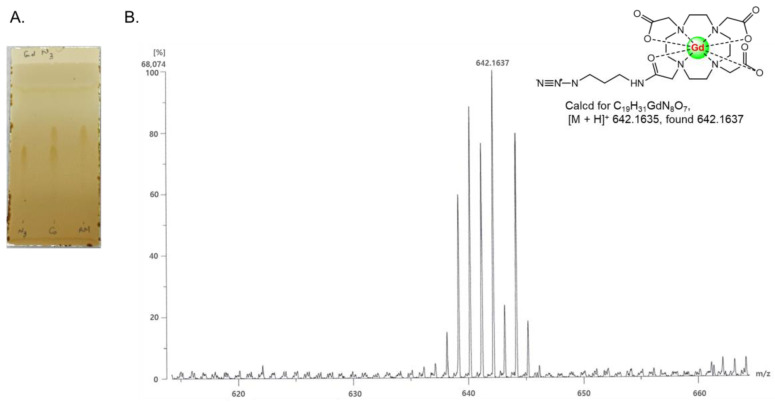
Thin-layer chromatography (TLC) to follow the reaction of Gd-azido-DOTA (**A**) (N_3_, azido-DOTA; Co, combined spots azido-DOTA and reaction mass; RM, reaction mass). HR-FAB mass spectrum of Gd-azido-DOTA (**B**).

Procedure 1 failed in the synthesis of the designed probe. Therefore, procedure 2, which included two synthesis steps, was conducted by making the Gd-azido-DOTA complex first; then, a click chemistry reaction between SF-alkyne and Gd-azido-DOTA was achieved. In the first step, the Gd-azido-DOTA complex was obtained as a white solid and quantality. According to C18 TLC (Figure 1A), the spot of the Gd-azido-DOTA (R_f_ of 0.64) product moved a little higher than the spot of azido-DOTA (R_f_ of 0.55), because in stationary reserve phase C18, more polar molecules tend to be less adsorbed onto the hydrophobic C18 stationary phase. The combined spots show two distinct spots that matched the azido-Dota and reaction mass spots. The nuclear magnetic resonance (NMR) spectrum of this Gd complex was not recorded on account of its paramagnetic property. The molecular weight of the Gd-azido-DOTA complex was confirmed via HR-FAB mass spectrometry. Mass calculated for C_19_H_31_GdN_8_O_7_ [M + H]^+^: 642.1635; found: 642.1637 (Figure 1B). In detail, the observed m/z values were 638.1496 (14.93%), 639.1529 (59.62%), 640.1563 (88.26%), 641.1600 (76.40%), 642.1637 (100.00%), 643.1616 (23.58%), 644.1657 (79.57%), 645.1668 (18.50%), 677.1231 (21.77%), 678.1223 (29.10%), 679.1111 (27.19%), 680.1196 (39.74%), and 682.1286 (27.83%). 

**Figure 2:**
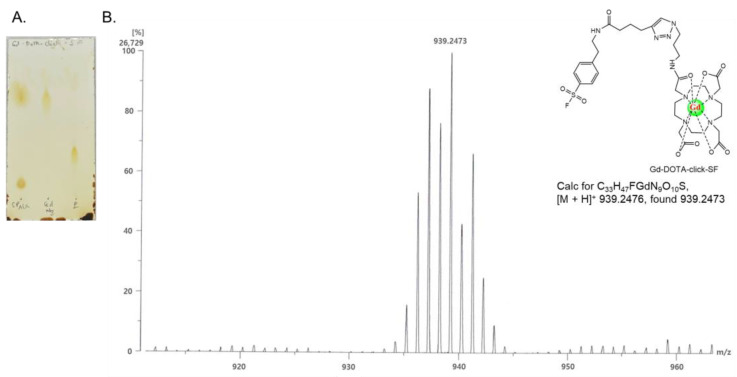
Thin-layer chromatography (TLC) of Gd-DOTA-click-SF after purification (**A**) (SF-Alk, SF-alkyne; Gd-N_3_, Gd-azido-DOTA; P, product (Gd-DOTA-click-SF after purification)). HR-FAB mass spectrum of Gd- DOTA-click-SF (**B**).

In the second step, the click reaction between Gd-azido-DOTA and SF-alkyne was carried out in a reducing agent (sodium ascorbate), CuSO_4_, and TBTA (Cu^I^-stabilizing ligand) at room temperature for 24 h. The final product was purified using a Sep-Pak plus short C18 cartridge. According to C18 TLC (Figure 2A), the product was pure and located between the SF-alkyne and Gd-azido-DOTA positions, because it was more polar than SF-alkyne and less polar than Gd-azido-DOTA. The product displayed a single spot at the retention factor (R_f_) of 0.4, while the SF-alkyne and Gd-azido-DOTA spots were observed at the retention factors (R_f_) of 0.17 and 0.79, respectively. In the second step, the final product was obtained as a pale-blue solid (29.8 mg; 48%). The product was then characterized using HR-FAB mass spectrometry. Mass calculated for C_33_H_47_FGdN_9_O_10_S [M + H]^+^: 939.2476; found: 939.2473 (Figure 2B). In particular, the observed m/z values were 935.2336 (15.93%), 936.2384 (88.07%), 938.2462 (76.61%), 939.2473 (100.00%), 940.2417 (42.88%), 941.2516 (66.42%), and 942.2547 (25.04%). The purity of the final product was checked using analytical high-performance liquid chromatography (HPLC) (Water Alliance 2695; Waters Corporation, Milford, MA, USA) with a photodiode array detector (PDA; Waters 2996). According to the HPLC/PDA result, the purity of our product (peak at 9.624 min; wavelength of 228 nm) was more than 97% (Figure S2A). The main peak was confirmed again using UPLC/TOF-MS. According to the result, the two precursors, SF-alkyne and Gd-azido-DOTA, were not observed in the desired product. The desired product appeared as the major peak. Mass calculated for C_33_H_47_FGdN_9_O_10_S [M + H]^+^: 939.2476; found: 939.248 (Figure S2B). 

### 2.2. Relaxivity Measurements

**Figure 3:**
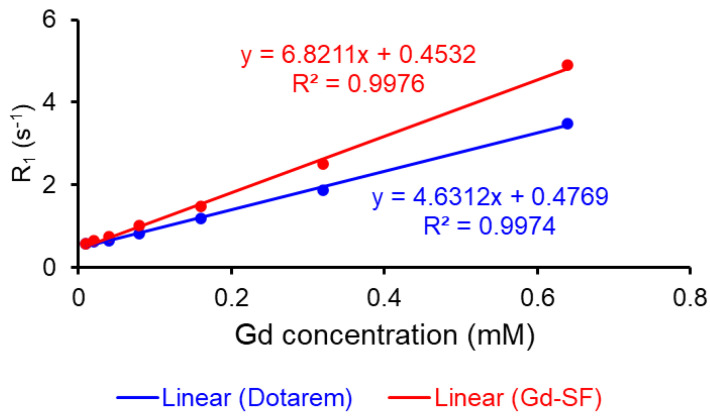
Plots of relaxation rate as a function of gadolinium concentration (0.01, 0.02, 0.04, 0.08, 0.16, 0.32, and 0.64 mM Gd). The slopes correspond to the molar longitudinal relaxivity (r_1_) of Dotarem (r_1_ = 4.63 mM^−1^ s^−1^) and Gd-DOTA-click-SF (r_1_ = 6.82 mM^−1^ s^−1^) obtained using the inversion recovery method at 9.4 T.

To obtain quantitative information on the effectiveness of the Gd-DOTA-click-SF probe and Dotarem as MRI contrast agents, we determined their molar longitudinal relaxivity (r_1_) at 3.0 T, 22.3 °C, using the inversion-recovery method, or 9.4 T, 19 °C, using the RARE method. With 3.0 T, the results show that the molar longitudinal relaxivity (r_1_) of the Gd-DOTA-click-SF probe (r_1_ = 7.04 mM^−1^ s^−1^) was higher than that of Dotarem (r_1_ = 3.99 mM^−1^ s^−1^) due to the increased molecular weight of the Gd-DOTA-click-SF probe [24,30] (Figure S3). This result is in agreement with that obtained with 9.4 T equipment. When using the 9.4 T MRI machine, the molar longitudinal relaxivity r_1_ of the Gd-DOTA-click-SF probe (r_1_ = 6.82 mM^−1^ s^−1^) was higher than that of Dotarem (r_1_ = 4.63 mM^−1^ s^−1^) in the range of concentrations from 0.01 to 0.64 mM (Figure 3). The summary of the results is displayed in Table 1.

### 2.3. Transmetallation Kinetic Study

**Figure 4:**
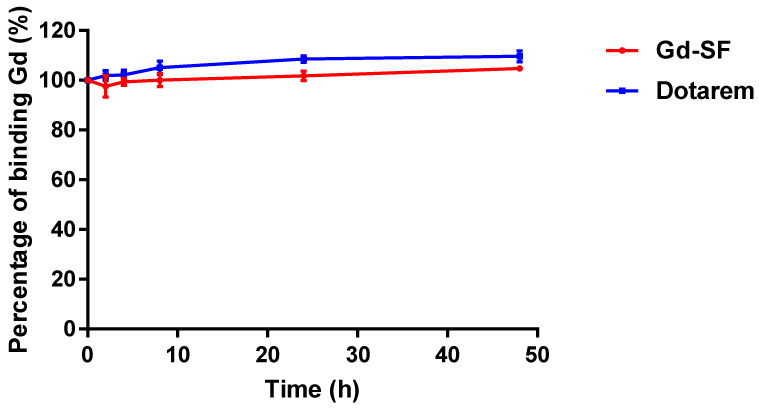
Gd(III) content in Gd-DOTA-click-SF and Dotarem at different time points of incubation in PBS buffer and ZnCl_2_ (250 mM).

Gd ions are highly toxic in free form because they are insoluble at physiological pH, leading to very slow systemic excretion, with the majority being deposited in bone, kidney, and liver, causing nephrotoxicity and neurotoxicity. In vivo, the transmetallation of the Gd complex occurs due to Gd^3+^ ions competing with various endogenous cations and endogenous anions, which destabilizes the Gd complex and shifts the dissociation equilibrium toward its free components [14,22,23]. Therefore, the in vitro stability of the Gd(III) complex needs to be investigated in the presence of Zn(II) ions. As the results show, the stability of Gd-DOTA-click-SF is comparable to that of Dotarem. Both complexes showed high stability, without transmetallation occurring in the presence of Zn(II) ions (Figure 4).

### 2.4. In Vitro Cell Viability Test

**Figure 5:**
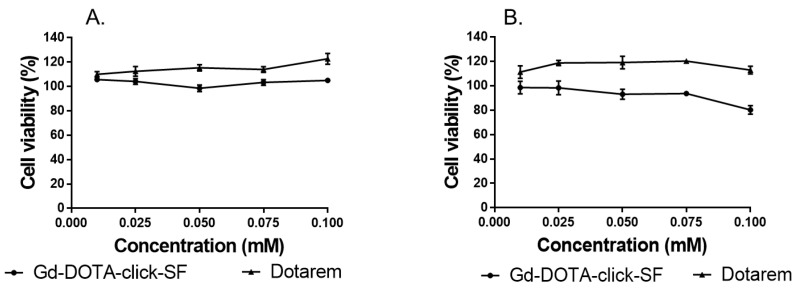
Cell viability CCK-8 assay with two different cell lines after 24 h of incubation: RAW 264.7 cells (**A**) and C166 endothelial cells (**B**).

The cytotoxicity of Gd-DOTA-click-SF was evaluated using CCK-8 assays with RAW 264.7 cells and endothelial C166 cells. The viability values of these cells showed no significant changes and were comparable to those recorded with Dotarem (Figure 5). No significant toxicity was observed with any of the compounds. 

### 2.5. MALDI-TOF/TOF Mass Spectrometry Shows That Gd-DOTA-Click-SF Probe Specifically Binds to Serine Proteases 

**Figure 6:**
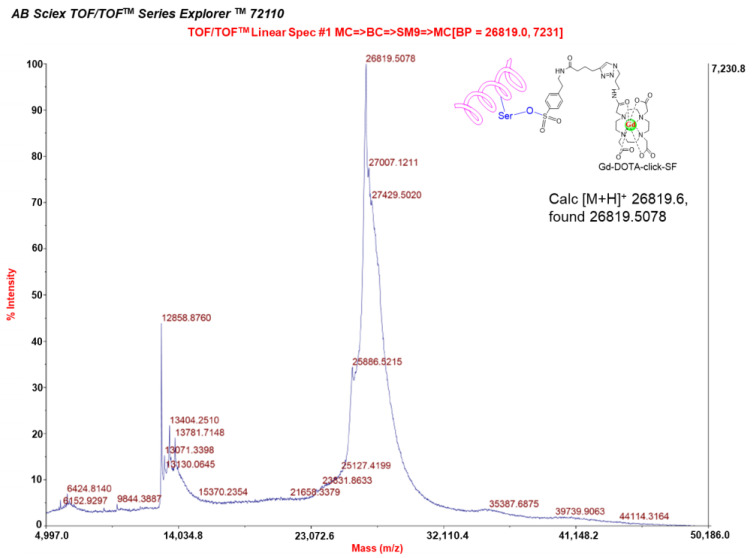
MALDI-TOF/TOF mass spectrum of Gd-DOTA-click-elastase (linear mode).

As the results show, the formation of Gd-DOTA-click-elastase demonstrated the labeling of the entire elastase present (which belongs to the serine proteases family) by Gd-DOTA-click-SF. The molecular weight of Gd-DOTA-click-elastase was 26,819.5078 Da, which matches the theoretical molecular weight of 26,819.6 Da (Figure 6). The results obtained suggest that the other peptides (such as somatostatin and oxytocin) and the protein (ribonuclease A) did not react with Gd-DOTA-click-SF. The intact molecular weights of Gd-DOTA-click-SF (939.2476), these peptides (somatostatin (1637.88) and oxytocin (1007.19)), and protein (ribonuclease A (13,683.30)) remained discernible (Figures S5–S8, respectively). These results are in agreement with those in reference [25]. 

### 2.6. Ex Vivo T1-Weighted MRI for Tracking Elastase Activity in Experimentally Induced Rat AAA Development 

**Figure 7:**
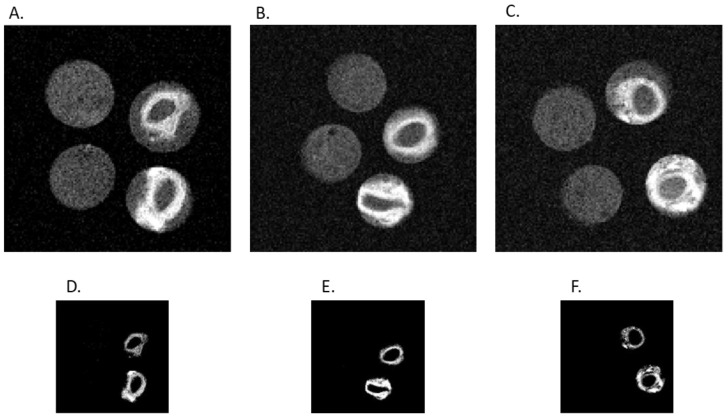
T1-weighted MR images of healthy abdominal artery (H) and abdominal artery aneurysm (AAA) immersed in Dotarem and Gd-SF (H-Do, AAA-Do, H-Gd-SF, and AAA-Gd-SF). ((**A**,**D**), Lot 1; (**B**,**E**), Lot 2; (**C**,**F**), Lot 3. *n* = 3. Top left, H-Do; top right, H-Gd-SF; bottom left, AAA-Do; bottom right, AAA-Gd-SF. (**D**–**F**) brightness/contrast adjustment).

To evaluate the potential of the Gd-SF probe as an elastase activity sensing probe for rat AAA visualization, we obtained ex vivo MRI scans of four different groups, including healthy rat aorta immersed in Dotarem (H-Do) and Gd-SF (H-Gd-SF) solutions (1.28 mM), and rat AAA immersed in Dotarem (AAA-Do) and Gd-SF (AAA-Gd-SF) solutions (1.28 mM). Figure 7 shows that the highest T1- signal intensity was observed in AAA-Gd-SF specimens. H-Do and AAA-Do specimens did not show high signal intensity in T1-weighted MRI, which means that Dotarem was eluted during the washing step and could not bind to the arterial walls. H-Gd-SF also showed high T1 signal intensity but lower than that of AAA-Gd-SF due to the non-specific binding to the arterial walls. 

### 2.7. Comparisons of the CNRs of T1-Weighted MRI and of Gadolinium Concentration Measurements Using ICP/MS among Four Different Study Groups 

**Figure 8:**
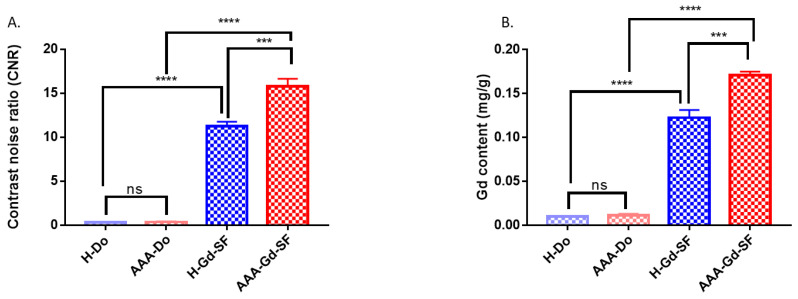
Comparison of the CNRs of the T1-weighted MR images among the four groups of rat healthy abdominal aorta and abdominal aortic aneurysm immersed in either Dotarem or Gd-SF (H-Do, AAA-Do, H-Gd-SF and AAA-Gd-SF) (**A**). Comparison of gadolinium concentrations accumulated in healthy abdominal aorta and abdominal aortic aneurysm immersed in either Dotarem or Gd-SF (H-Do, AAA-Do, H-Gd-SF, and AAA-Gd-SF) using ICP/MS measurements (**B**). (*n* = 3 per study group. One-way ANOVA; ns, not significant; *** *p* < 0.001, and **** *p* < 0.0001).

According to Figure 8A, the CNR of AAA-Gd-SF was significantly different from those of the other groups. This result shows good agreement with the ICP/MS results (Figure 8B). These results prove the concept that Gd-SF specifically binds to elastase to enhance the signal intensity in the T1-weighted MRI of rat AAA models. 

## 3. Discussion

Despite the strong connection between serine protease and numerous disorders, there have been few clinically available imaging methods to demonstrate its enzymatic activity. MRI is a widely used, non-invasive, and non-ionizing diagnostic imaging modality. Gadolinium-based contrast agents are often used to improve image contrast. Conventional Gd chelates are non-targeted contrast agents with low specificity. Herein, we designed and synthesized Gd-DOTA-click-SF (Gd-SF), which can target serine proteases such as elastase thanks to the covalent and specific binding between the sulfonyl fluoride (SF) group and the hydroxy (OH) group of the serine residue in the active site. The DOTA backbone was chosen because it is considered the “gold standard” ligand, with high stability in trivalent gadolinium chelation. It is believed to be the most thermodynamically stable GBCA in clinical use, as it is N-functionalized, with four acetic acid pendant arms, and is octodentate, with four nitrogen and four oxygen donor atoms, allowing it to coordinate with the Gd atom [31]. This DOTA precursor has an azide reaction group, which is used for the click reaction with the alkyne contained in the sulfonyl fluoride (SF) group. The sulfonyl fluoride (SF) group of the probe reacts with the hydroxy group (OH) of serine proteases. 

Our first synthesis procedure failed due to the complex formation of catalysts Cu(II) and DOTA. However, our synthesis procedure was successful on the second attempt and included a two-step reaction, with 48% overall yield: In the first step, Gd reacted with azido DOTA to form the Gd-azido-DOTA complex, and the final step is the well-known azide-alkyne click reaction to obtain a nine-coordinate complex, the Gd-DOTA-click-SF probe. The successful formation of our designed chelate was confirmed with HR-FAB mass spectrometry. The starting material, azido-mono-amide-DOTA (azido DOTA), was allowed to react with GdCl_3_ at 60 °C for 24 h at pH = 5.5, and free Gd was removed using chelex^®^100 resin. According to C18 TLC (Figure 1A), the spot of the Gd-azido-DOTA product (R_f_ of 0.64) moved a little higher than the spot of azido-DOTA (R_f_ of 0.55), because in stationary reserve phase C18, more polar molecules tend to be less adsorbed onto the hydrophobic C18 stationary phase. HR-FAB mass spectrometry showed the successful synthesis of Gd-azido-DOTA. Mass calculated for C_19_H_31_GdN_8_O_7_ [M + H]^+^: 642.1635; found: 642.1637 (Figure 1B). In the second step, the click reaction between Gd-azido-DOTA and SF-alkyne was carried out in a reducing agent (sodium ascorbate), CuSO_4_, and TBTA (Cu^I^-stabilizing ligand) at room temperature for 24 h. The final product was purified with a Sep-Pak plus short C18 cartridge. According to the C18 TLC (Figure 2A) and HPLC/PDA results (Figure S2A), the product was pure (more than 97%) and located between the SF-alkyne and Gd-azido-DOTA positions, because Gd-DOTA-click-SF (R_f_ of 0.4) is more polar than SF-alkyne (R_f_ of 0.17) and less polar than Gd-azido-DOTA (R_f_ of 0.79). The synthesis of this novel Gd-DOTA-click-SF probe was then characterized using HR-FAB mass spectrometry. Mass calculated for C_33_H_47_FGdN_9_O_10_S [M + H]^+^: 939.2476; found: 939.2473 (Figure 2B). 

The Gd-DOTA-click-SF probe exhibited an exceptional increase in relaxivity (r_1_) at both 3.0 T and 9.4 T, with values of 7.04 mM^−1^ s^−1^ and 6.82 mM^−1^ s^−1^, respectively. Notably, these values significantly surpassed those of Dotarem, which recorded values of 3.99 mM^−1^ s^−1^ and 4.63 mM^−1^ s^−1^ at 3.0 T and 9.4 T, respectively. The remarkable improvement in relaxivity of the Gd-DOTA-click-SF probe can be attributed to its greater molecular weight, highlighting its superiority over Dotarem [13,24] (Table 1). 

The dissociation of Gd ions from their chelating ligands causes the potential toxicity of Gd chelates. Moreover, in vivo, the transmetallation of the Gd complex occurs due to Gd^3+^ ions competing with different endogenous cations for the ligand, which destabilizes the gadolinium complex. Therefore, we examined the stability of our probe with a transmetallation study. According to our results, the stability of the synthesized Gd(III) complex was comparable to that of Dotarem (Figure 4).

The cytotoxicity of Gd-DOTA-click-SF was evaluated using CCK-8 assays with RAW 264.7 cells and endothelial C166 cells. The viability values of these cells showed no significant changes and were comparable to those recorded with Dotarem (Figure 5). No significant toxicity was observed with these compounds.

The advantage of our probe is the inclusion of the sulfonyl fluorides (SF) targeted group. This functional group is known to specifically react with the nucleophilic hydroxy (OH) group serine residue in the active site. This new-generation click chemistry-sulfonyl fluoride electrophile exchange is used in the development of reactive probes in chemical biology and medicinal chemistry. To evaluate the ability of the Gd-DOTA-click-SF probe to image serine proteases such as elastase, we firstly confirmed the click reaction of the OH group of the serine residue, such as elastase, covalently binding to the Gd-DOTA-click-SF probe using MALDI-TOF/TOF mass spectrometry. 

As a result, the formation of Gd-DOTA-click-elastase demonstrated the labeling of the entire elastase present by Gd-DOTA-click-SF; the molecular weight of Gd-DOTA-click-elastase was 26,819.5078 Da, which matches the theoretical molecular weight of 26,819.6 Da (Figure 6). The other peptides and the protein did not react with Gd-DOTA-click-SF, as shown in Figures S6–S8. The intact molecular weight was still discernible. These results are in agreement with those of reference [25].

After the in vitro MALDI-TOF/TOF mass spectrometry study, we extended the preliminary application of our probe to ex vivo AAA MRI. AAA is a widespread and potentially fatal vascular disease. Current techniques used to detect AAAs are ultrasound, PET, CT, and MRI. Elastase levels have been found to be high in ruptured aneurysms [6,9]. Elastase, which can be used as an inflammatory marker, is an enzyme belonging to the serine protease family. Our probe was proven to have reacted with elastase based on the sulfonyl fluorides (SF) group. According to the ex vivo results (Figure 7), the AAA-Gd-SF specimens showed the highest T1-weighted signal intensity. There was no high T1-weighted signal intensity in the T1-weighted MRI of H-Do and AAA-Do specimens, which means that Dotarem was eluted during the washing step and could not bind to the arterial vessel walls. H-Gd-SF also showed high T1 signal intensity, but lower than AAA-Gd-SF due to the non-specific binding to the arterial vessel walls. Moreover, the CNR of AAA-Gd-SF was significantly different from those of other groups (Figure 8A), which is in good agreement with the ICP/MS results (Figure 8B). Taken together, these results prove the concept that Gd-SF specifically targets and binds to elastase to enhance the signal intensity in the T1-weighted MRI of AAA models.

Although our current study obtained the expected results, there are still some limitations. Firstly, we could not perform in vivo AAA imaging experiments, which will be our target in future research. For newly developed contrast agents to be used in clinical imaging, their biosafety in animal models must be considered. In the current study, the novel contrast agent provided the initial basis for use in clinical imaging, such as cell cytotoxicity and probe stability. It is known that the toxicity of Gd complex contrast agents is due to the dissociation of Gd ions from chelating ligands [16,17,18,19,20,21]. Gd in free form is insoluble at physiological pH, leading to very slow systemic excretion, with the majority being deposited in bone, kidney, and liver, which can cause nephrotoxicity. However, the DOTA backbone shows high stability (logK = 25.3) and is safe because the central Gd metal binds to the water molecules and the octagonal coordination while maintaining kinetic inertia and dynamic stability [24]. The DOTA backbone is currently regarded to be highly stable and safe; therefore, it was selected in this study. Our study shows that the stability of Gd-DOTA-click-SF is comparable to that of Dotarem. Both complexes showed high stability without transmetallation in the presence of Zn(II) ions (Figure 4). Secondly, although the r_1_ relaxivity of the new contrast agent Gd-SF was compared to that of Dotarem at two different field strengths (clinical 3.0 T and pre-clinical 9.4 T) to establish the effectiveness of Gd-SF, perfect agreement of the experimental conditions could not be achieved at the two magnetic fields. The r_1_ values can be affected by the magnetic field, concentration, temperature, and solvent [32,33,34,35,36]. Therefore, further investigations are required to fully understand the factors impacting r_1_ relaxivity of Gd-SF.

## 4. Materials and Methods

### 4.1. Materials and Reagents

4-(2-(Hex-5-ynamido)ethyl)benzenesulfonyl fluoride (SF-alkyne), Copper(II) sulfate pentahydrate (CuSO_4_·5H_2_O), sodium ascorbate, Tris[(1-benzyl-1H-1,2,3-triazol-4-yl)methyl]amine (TBTA), ammonium acetate (CH_3_COONH_4_), N,N-Dimethylformamide (DMF), tert-butanol, iodine (I_2_), gadolinium (III) chloride hexahydrate 99.999% on a trace metal basis (GdCl_3_·6H_2_O), tris(hydroxymethyl)aminomethane hydrochloride (Tris-HCl), chelex^®^ 100 sodium form, and elastase (E7885; from porcine pancreas) were purchased from Sigma Aldrich (St. Louis, MO, USA). 1,4,7,10-Tetraazacyclododecane-1,4,7-tris acetic acid-10-(azidopropyl-ethylacetamide) (azido-mono-amide-DOTA) was purchased from Macrocyclics (Dallas, TX, USA). Gadoterate meglumine (Dotarem^®^, Guerbet, Aulnay-sous-Biois, France) was purchased from Guerbet. Methanol (MeOH) and potassium hydroxide (KOH) were purchased from Duksan. Sep-Pak plus short C18 (Waters Corporation, Milford, MA, USA) was used for the purification of Gd-DOTA-click-SF. HyClone Dulbecco’s Modified Eagle Medium (DMEM; Cytiva, Marlborough, MA, USA) with high glucose was used. Fetal bovine serum (FBS; Gibco, Thermo Fisher Scientific, Waltham, MA, USA), Penicillin-Streptomycin (10,000 U/mL) (Thermo Fisher Scientific, Waltham, MA, USA), and cell counting kit-8 (CCK-8; Dojindo Molecular Technologies, Rockville, MD, USA) were employed. All commercially available reagents and chemicals were used as received without further purification.

Thin-layer chromatography (TLC) silica gel 60 RP-18 F_254_S was purchased from Merck.

Ultrapure water with resistivity of 18.2 mΩ·cm at 25 °C was obtained using a Milli-Q water purification system (Millipore Corporation, Burlington, MA, USA) and used throughout the study.

Chemical structures were drawn using ChemDraw version 18.0 software.

GraphPad Prism 7.02 (GraphPad Software Inc., San Diego, CA, USA) software was used for figure and statistical analyses. *p*-values < 0.05 were considered statistically significant.

### 4.2. Instrumentation and Characterization 

MRI experiments were conducted at 3.0 T (GE Healthcare, SIGNA Architect, Chicago, IL, USA) and 9.4 T (BioSpec 94/20 USR; Bruker BioSpin, Ettlingen, Germany). Low resolution and high resolution with fast atom bombardment (FAB) ionization (JMS 700 Jeol; Akishima, Tokyo, Japan) was used for the characterization of Cu-DOTA-click-SF, Gd-azido-DOTA, and Gd-DOTA-click-SF formations. Matrix-assisted laser desorption/ionization coupled to time-of-flight mass spectrometry (MALDI-TOF MS) (MALDI-TOF/TOF^TM^ 5800 system; AB Sciex) was used for the confirmation of the reaction between elastase and Gd-DOTA-click-SF. A SpectraMax^®^ 190 absorbance microplate reader (Molecular Devices, San Jose, CA, USA) was used. Inductively coupled plasma coupled to mass spectrometry (ICP/MS; 7700; Agilent, Santa Clara, CA, USA) was used. 

### 4.3. Synthesis of Gd-DOTA-Click-SF Probe with Click Chemistry

#### 4.3.1. Procedure 1: Synthesis of DOTA-Click-SF with Click Chemistry

**Scheme 1:**
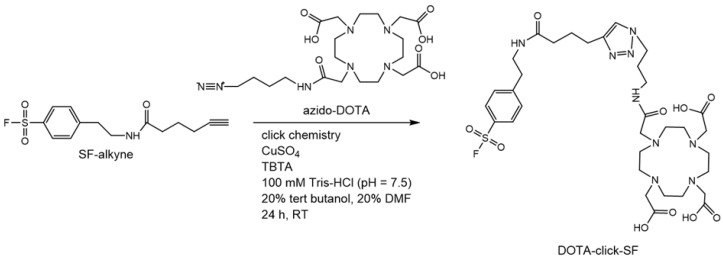
Schematic illustration of the synthesis procedure of DOTA-click-SF achieved by coupling SF-alkyne and azido-DOTA with click chemistry.

The click chemistry reaction conditions between azido-mono-amide-DOTA and SF-alkyne were set according to the literature, with minor modifications [18]. Azido-mono-amide-DOTA (6 mg; 0.01 mmol) and SF-alkyne (4.5 mg; 0.015 mmol; 1.5 eq) (Scheme 1) were mixed in 5 mM sodium ascorbate (reducing reagent), 1 mM CuSO_4_ (catalyst), 0.1 mM TBTA (Cu^I^-stabilizing ligand [37]), 100 mM Tris-HCl (pH = 7.5), and 20% tert-butanol. The resulting reaction mixture was stirred at room temperature for 24 h. The formation of DOTA-click-SF was checked with thin-layer chromatography (TLC) on C18 silica plates with a suitable mobile phase (MeOH:10% CH_3_COONH_4_ (1:1)) and was visualized using iodine staining. The mixture was then concentrated under reduced pressure and dissolved in water.

#### 4.3.2. Procedure 2: Synthesis of Gd-DOTA-Click-SF Probe Using Gd-Azido-DOTA and SF-Alkyne with click Chemistry

##### Step 1: Synthesis of Gd-Azido-DOTA

**Scheme 2:**
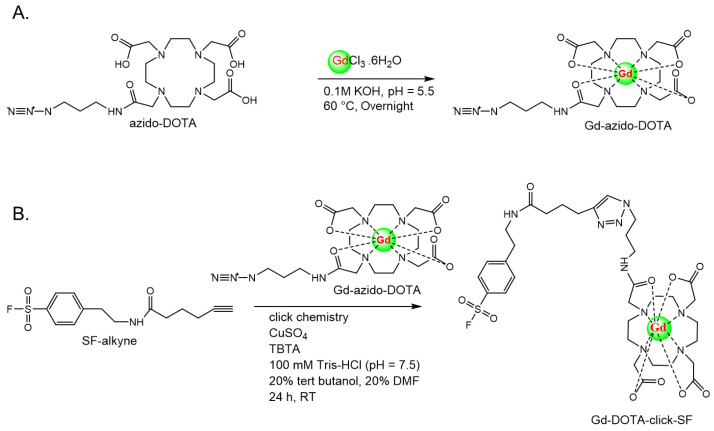
Schematic illustration of the synthesis procedure of Gd-azido-DOTA complex (**A**) and Gd-DOTA-click-SF probe (**B**).

To a solution of azido-mono-amide-DOTA (38.0 mg; 0.064 mmol; 1 eq) in aqueous deionized water (DI H_2_O), 500 µL of GdCl_3_·6H_2_O (30.8 mg; 1.3 eq) was added, and the pH was adjusted to 5.5 with 0.1 M KOH solution (Scheme 2A). The mixture was then heated to 60 °C for 24 h under stirring [24]. The formation of the gadolinium complex was monitored with thin-layer chromatography (TLC) on C18 silica plates (Figure 1A). The TLC plates were developed in the mobile phase (MeOH:10% CH_3_COONH_4_ (1:2)). The mixture was then concentrated using a rotary evaporator and dissolved in DI H_2_O. Free Gd^3+^ ions were removed by adding chelex^®^100 resin to the solution, and the solution was gently stirred for 2 h; then, the supernatant was decanted and concentrated using a rotary evaporator. 

##### Step 2: Synthesis of Gd-DOTA-Click-SF Probe [25]

The click chemistry reaction conditions for Gd-azido-DOTA (42 mg; 0.065 mmol; 1.1 eq) and SF-alkyne (18 mg; 0.06 mmol; 1 eq) (Scheme 2B) included sodium ascorbate (57.2 mg; 4.8 eq), CuSO_4_·5H_2_O (15 mg; 1 eq), TBTA (32 mg; 1 eq), 100 mM Tris-HCl (pH = 7.5), 20% tert-butanol, and 20% DMF. The resulting reaction mixture was stirred at room temperature for 24 h. The formation of Gd-DOTA-click-SF was checked with thin-layer chromatography (TLC) on C18 silica plates. The TLC plates were developed in mobile phase (MeOH:10% CH_3_COONH_4_ (1:1)), and the spots were visualized using iodine staining. The mixture was then concentrated using a rotary evaporator and dissolved in DI H_2_O. The crude compound was isolated using Sep-Pak plus short C18 purification. The following are the detailed purification steps: At first, the stationary phase of the C18 cartridge was solvated with methanol (MeOH) and then flushed with HPLC-grade water. Next, the crude Gd-DOTA-click-SF in HPLC-grade water was loaded onto the cartridge. A series of washes were performed with HPLC-grade water to remove the unreacted reagents, such as reducing agent (sodium ascorbate), CuSO_4_, and TBTA (Cu^I^-stabilizing ligand). Afterwards, the compound was eluted with MeOH:10% CH_3_COONH_4_ (1:4) in approximately 50 mL. The collected fractions were confirmed using TLC analysis (C18 TLC; MeOH:10% CH_3_COONH_4_ (1:1)) (Figure 2A). The product displayed a single spot at the retention factor (Rf) of 0.4, while SF-alkyne and Gd-azido-DOTA showed spots at the retention factors (Rf) of 0.17 and 0.79, respectively. The retention factor (Rf) is the ratio of the distance that a spot has traveled to the distance that the solvent has traveled. The synthesized Gd-DOTA-click-SF probe was then characterized using HR-FAB mass analysis. Mass calculated for C_33_H_47_FGdN_9_O_10_S [M + H]^+^: 939.2476; found: 939.2473 (Figure 2B). 

### 4.4. Relaxivity Measurements of Gd-DOTA-Click-SF Probe [38,39]

The relaxation times and map images were measured with a magnetic resonance imaging (MRI) instrument at 3.0 T (GE Healthcare, SIGNA Architect, Chicago, IL, USA) equipped with a head coil at 22.3 °C and 9.4 T (BioSpec 94/20USR; Bruker BioSpin, Ettlingen, Germany) at 19 °C to test the Gd-DOTA-click-SF probe, which was used as an MRI contrast agent. With 3-Tesla equipment, the following parameter values were utilized: static echo time = 24 ms; field of view (FOV) = 20 × 20 mm^2^; matrix size = 320 × 160; number of axial slices = 3; slice thickness = 5.0 mm. In order to measure the T1 relaxation time, the inversion recovery method was used. The inversion time (TI) sequence was set to 1700, 100, 1600, 200, 1500, 300, 1400, 400, 1300, 500, 1200, 600, 1100, 700, 1000, 800, and 900. This procedure was repeated for designated concentrations of Gd-DOTA-click-SF (0.01, 0.02, 0.04, 0.08, 0.16, 0.32, and 0.64 mM). Samples of the probe were prepared by diluting 50.3 mM stock solution. The stock Gd (III) concentration was precisely determined using inductively coupled plasma mass spectrometry (ICP/MS; 7700; Agilent, USA). MR phantom images were obtained with 1.5 mL Eppendorf vials. Dotarem was chosen as a commercial clinical control. The R_1_ relaxation rate was calculated using a single exponential fitting of the MR signal at different inversion times with the following equation [40,41]:

SIR (TI) = A × [1 − 2 × exp (−R_1_·TI)]

where parameter SIR represents the MRI signal intensity, TI is the inversion time, and r_1_ is calculated according to the slope of the 1/T1 (R_1_) plots versus Gd (III) ion concentrations.

With 9.4 T equipment, MR phantom images were obtained using phantom wells used for measuring relaxivity according to the concentration. The test sample solution in the phantom well was imaged on the mouse bed and fixed so that it did not move. The MRI coil used was an 84 mm volume coil for scanning. The area was scanned using the localizer, and the inversion recovery RARE (rapid acquisition with refocused echoes) sequence was used for measuring T1. RARE is a technique for obtaining a spin echo image within a short time by obtaining echoes having different phase codes from 2 to 16 times during one repetition time (TR) and filling the k-space. The MRI parameters were as follows: TE = 10 ms; TR = 10,000 ms; the inversion time (TI) sequence set to 85, 140, 200, 300, 500, 700, 1000, 2000, 3000, 5000, and 7000 ms; rare factor = 1; FOV = 50 × 50 × 5 mm^3^; matrix size = 128 × 128 × 5; slice orientation = coronal. 

The concentrations of Gd-DOTA-click-SF and Dotarem were used in the same range (0.01, 0.02, 0.04, 0.08, 0.16, 0.32, and 0.64 mM) with 3.0 T.

### 4.5. Transmetallation Kinetics Study [13]

Briefly, 8 µL of 250 mM aqueous solution of ZnCl_2_ was added into 1.5 mL of 1 mM Gd-DOTA-click-SF complex in phosphate buffer. The mixture was stirred. Then, the solution was collected at each time point (0, 2, 4, 6, 8, 24, and 48 h) and filtered. The concentration of Gd-DOTA-click-SF was measured with ICP/MS. The percentage of bound Gd(III) post-incubation with respect to the value before incubation demonstrated kinetic inertness. In order to compare the stability with that of the clinical contrast agent Dotarem, the same procedure was conducted. The experiment was repeated 3 times.

### 4.6. In Vitro Cell Viability Test 

The cytotoxicity of the Gd-DOTA-click-SF probe was assessed against macrophage raw 264.7 cells and endothelial C166 cells using a CCK-8 assay according to the instructions provided by the manufacturer. Cells were grown in DMEM complete medium containing 10% FBS and 1% Penicillin–Streptomycin (10,000 U/mL). RAW 264.7 cells were seeded on a 96-well plate at 2.5 × 10^4^ cells per well in 100 µL of DMEM completed medium. After 24 h of incubation at 37 °C in a humidified atmosphere containing 5% CO_2_, the cells were supplemented with Gd-DOTA-click-SF probe and Dotarem at different concentrations (0.01, 0.025, 0.05, 0.075, and 0.1 mM) for comparison and were incubated for 24 h at 37 °C. Afterwards, the cells were treated with 10 µL of CCK-8 solution. After further incubation for 2 h at 37 °C in an incubator, the absorbance of the wells was read at 450 nm using a microplate reader. The percentage of cell viability at various Gd-DOTA-click-SF probe and Dotarem concentrations was determined as the ratio of the absorbance of the sample versus that of the control:

$$\mathrm{Cell}\;\mathrm{viability}\;\left(\%\right)=\left[\frac{{\mathrm{Abs}}_{\mathrm{sample}}-{\mathrm{Abs}}_{\mathrm{blank}}}{{\mathrm{Abs}}_{\mathrm{control}}-{\mathrm{Abs}}_{\mathrm{blank}}}\right]$$

where Abs_sample_ is the absorbance of the test sample, Abs_control_ is the absorbance of the control sample, and Abs_blank_ is the absorbance of the blank sample.

The viability experiments were performed in triplicate (*n* = 3). This test procedure was also applied for C166 cells, with the only differences being the cell density (3 × 10^3^ cells/well) and the CCK-8 incubation time (4 h).

### 4.7. MALDI-TOF/TOF Mass Spectrometry Shows That Gd-DOTA-Click-SF Probe Specifically Binds to Serine Proteases

To examine the specific targeting of serine proteases, we investigated the reaction between Gd-DOTA-click-SF (160 µM) and elastase (which belongs to the serine protease family), as well as between Gd-DOTA-click-SF and other peptides (oxytocin and somatostatin), and ribonuclease A protein at the same concentration, with 100 mM Tris-HCl (pH = 7.5), and incubated the samples at 37 °C for 1 h prior to MALDI-TOF mass spectrometry. Detailed instrument settings and mass calibration are available in the supporting information (Table S1 and Figure S4, respectively).

### 4.8. Ex Vivo T1-Weighted MRI Tracking Elastase to Visualize AAA Development

Animal care and all experimental procedures were approved and conducted in accordance with the guidelines of the Institutional Animal Care and Use Committee of Catholic University of Daegu (approval number: DCIAFCR-220104-26-Y).

Seven-week-old Sprague-Dawley (SD) male rats (Samtako, Osan, Republic of Korea) were maintained on a 12-hour light–dark cycle with free access to water and normal rodent chow for one acclimatization week prior to experiments. A total of 16 rats were randomly divided into 4 groups comprising healthy aorta immersed in Dotarem (H-Do) and Gd-SF (H-Gd-SF), and AAA immersed in Dotarem (AAA-Do) and Gd-SF (AAA-Gd-SF). The AAA rat models were created by performing elastase injection in the rat aorta according to the literature, with small modifications [7,8]. Briefly, SD male rats were anesthetized with 2 to 2.5% isoflurane inhalation, and their abdomen was shaved and scrubbed with alcohol and betadine. A long midline abdominal incision was made, and the infrarenal abdominal aorta was isolated under sterile conditions. The branches of the abdominal aorta were exposed and ligated with 10-0 sutures. All aortic branches within 1.0 cm of the bifurcation were ligated. Temporary 6.0 silk ligatures were placed around the proximal and distal portions of the aorta. The infrarenal abdominal aorta was cannulated with PE-10 tubing and perfused with 12 U porcine pancreatic elastase in a total volume of 300 μL with sterile normal PBS for 10 minutes. The perfusion catheter was then removed, and the aortotomy was closed. The intestines were replaced, and the abdominal wall was closed. After 5 weeks, the aorta was harvested, and the blood was removed. Afterwards, it was immersed in Dotarem or Gd-SF at 1.28 mM for 1 h under shaking. Then, it was taken out and washed 3 times with PBS. Finally, the specimens were put in PCR tubes filled with PBS and examined with T1-weighted MRI. The same procedure was also applied to healthy aorta specimens, which were immersed in Dotarem or Gd-SF at 1.28 mM.

Ex vivo T1-weighted MRI was performed at 9.4 T on a Bruker scanner using a 23 mm volume coil. A RARE sequence was used for image acquisition with the following parameters: echo time (TE) = 10 ms; rare factor = 1; repetition time (TR) = 300 ms; FOV = 10 × 10 × 10 mm^3^; matrix size = 128 × 128 × 10; slice orientation = coronal.

### 4.9. Determination of Gd Concentration with ICP/MS Measurement

Following T1-weighted MRI, the healthy abdomen aorta and aneurysm abdomen aorta specimens were digested in concentrated HNO_3_ acid, and we measured the concentration of Gd with ICP/MS (ICP/MS; 7700; Agilent, Santa Clara, CA, USA).

## 5. Conclusions 

To summarize, we successfully designed and synthesized the Gd-DOTA-click-SF probe, which not only has higher molar longitudinal relaxivity (r_1_) (r_1_ = 7.04 mM^−1^s^−1^ at 3 T, and 6.82 mM^−1^s^−1^ at 9.4 T) than Dotarem (r_1_ = 3.99 mM^−1^s^−1^ at 3.0 T, and 4.63 mM^−1^s^−1^ at 9.4 T) but also presents in vitro cellular biosafety and stability comparable to those of Dotarem. Moreover, thanks to the sulfonyl fluorides (SF) functional group with specific binding to arterial walls, experimentally induced rat abdominal aortic aneurysm with higher serine protease or elastase activity was proven in vitro using MALDI-TOF MS and preliminary ex vivo studies. In the near future, we will advance into pre-clinical and clinical trials to demonstrate that Gd-DOTA-click-SF might act as a noble MRI contrast agent probe, showing active serine protease or elastase binding to arterial walls in clinical applications beyond this ex vivo MRI examination. 

## Figures and Tables

**Table 1:** In vitro relaxivity values at 3.0 T, 22.3 °C and 9.4 T, 19 °C for Dotarem and Gd-DOTA-click-SF (Gd-SF).

Contrast Agents	MW (Da)	3.0 T, 22.3 °C r_1_ (mM^−1^ s^−1^)	9.4 T, 19 °C r_1_ (mM^−1^ s^−1^)
Dotarem	735.9	3.99	4.63
Gd-SF	939.2	7.04	6.82

Dotarem and Gd-DOTA-click-SF (Gd-SF) concentration: 0.01, 0.02, 0.04, 0.08, 0.16, 0.32, and 0.64 mM.

## Data Availability

Additional supporting data can be found in Supplementary Materials.

## Supplementary Materials

The following supporting information can be downloaded at: https://www.mdpi.com/article/10.3390/molecules28083538/s1, 1. Table S1. Operating information of MALDI-TOF/TOF for analysis of Gd-DOTA-click-SF and elastase. 2. Figure S1. Low-resolution FAB mass spectrum of Cu-DOTA click SF. 3. Figure S2. The purity of the final product was checked using HPLC/PDA (A) and mass confirmation was performed with UPLC/TOF-MS (B). 4. Figure S3. Plots of relaxation rate as a function of gadolinium concentration (0.01, 0.02, 0.04, 0.08, 0.16, 0.32, and 0.64 mM Gd). The slopes correspond to the molar longitudinal relaxivity (r_1_) of Dotarem (r_1_ = 3.99 mM^−1^s^−1^) and Gd-DOTA-click-SF (r_1_ = 7.04 mM^−1^s^−1^) obtained using the inversion recovery method. 5. Figure S4. MALDI-TOF/TOF mass calibration mix 3 in linear mode. 6. Figure S5. MALDI-TOF/TOF mass spectrum of Gd-DOTA-click-SF (reflector mode). 7. Figure S6. MALDI-TOF/TOF mass spectrum of Gd-DOTA-click-SF reaction with somatostatin (MW 1637.88) (reflector mode). 8. Figure S7. MALDI-TOF/TOF mass spectrum of Gd-DOTA-click-SF reaction with Oxytocin (MW 1007.19) (reflector mode). 9. Figure S8. MALDI-TOF/TOF mass spectrum of Gd-DOTA-click-SF reaction with ribonuclease A (MW 13683.30) (reflector mode). 10. Figure S9. Histopathologic analysis comparison of healthy control group and abdominal aortic aneurysm (AAA) group. Hematoxylin and eosin staining (A), diameter (B), Verhoeff–Van Gieson (C), elastin content (D), trichrome (E), and collagen content (F). Healthy group (*n* = 3) and AAA group (*n* = 6). Welch *t*-test, ** *p* < 0.01 and **** *p* < 0.0001. Scale bars: 100 µm (full aorta) and 50 µm (zoomed- in portion). 11. Figure S10. Correlation of ex vivo MRI and histopathology of ruptured rat AAA. (A) Picture of rat ruptured AAA. (B) Histopathological staining with hematoxylin and eosin for general morphology evaluation (B1), Verhoeff–Van Gieson for elastin (B2), and trichrome for collagen component identification (B3). (C) Ex vivo T1-weighted MR images of the same segment of rat ruptured AAA. Ref. [42] is cited in Supplementary Materials file.

## Sample Availability

Samples of the Gd-DOTA-click-SF compound are available from the authors.

## Abbreviations

GBCAs: gadolinium-based contrast agents; DTPA, diethylenetriaminepentaacetic acid; DOTA, 1,4,7,10-tetraazacyclododecane-1,4,7,10-tetraacetic acid; TBTA, Tris[(1-benzyl-1H-1,2,3-triazol-4-yl)methyl]amine; DMEM, Dulbecco’s Modified Eagle Medium; FBS, fetal bovine serum; CCK-8, cell counting kit-8; PET, positron emission tomography; CT, computed tomography; MRI, magnetic resonance imaging; T1: longitudinal relaxation time; R1: Relaxation rate corresponding to T1; FOV: field of view; NMR, nuclear magnetic resonance; ICP/MS, inductively coupled plasma coupled to mass spectrometry; MALDI-TOF MS, matrix-assisted laser desorption/ionization coupled to time-of-flight mass spectrometry; HR-MS, high resolution mass spectrometry; FAB, fast atom bombardment; TLC, thin layer chromatography; DMF, N,N-Dimethylformamide; PBS, phosphate buffered saline; SP, serine protease; CA, contrast agent; CNR, contrast to noise ratio.
