# Supplementary material for: Gadolinium-Cyclic 1,4,7,10-Tetraazacyclododecane-1,4,7,10-Tetraacetic Acid-Click-Sulfonyl Fluoride for Probing Serine Protease Activity in Magnetic Resonance Imaging
Source: Molecules. 2023 Apr 17;28(8):3538. doi: 10.3390/molecules28083538 (PMC10141219; doi:10.3390/molecules28083538)
Supplement: Supplementary file 1 [file molecules-28-03538-s001.zip › molecules-2236483-supplementary.pdf]

Supporting Information

Gadolinium-cyclic 1,4,7,10-tetraazacyclododecane-1,4,7,10-tetraacetic acid-click-Sulfonyl Fluoride for Probing Serine Protease Activity in Magnetic Resonance Imaging

Phuong Tu Huynh[1], Huy Duc Vu[1], Junghwa Ryu[1], Hee Su Kim[2], Hoesu Jung[3], Sung Won Youn[1*]

[1]Department of Radiology, Daegu Catholic University School of Medicine, 3056-6, Daemyung-4-Dong, Nam-gu, Daegu 705-718, Republic of Korea

[2]Korea Basic Science Institute (Daegu center), Kyungpook University, 80, Daehak-ro, Buk-gu, Daegu 41566, Republic of Korea

[3]Preclinical Research Center, Daegu-Gyeongbuk Medical Innovation Foundation (KMEDIhub), 88, Dongnae-ro, Dong-gu, Daegu 41061, Republic of Korea

*Correspondence Sung Won Youn, MD and PhD, Department of Radiology, Daegu Catholic University School of Medicine, 3056-6, Daemyung-4-Dong, Nam-gu, Daegu 705-718, Republic of Korea

Tel.: 82-53-650-4309

Fax: 82-52-650-4339

E-mail: ysw10adest@cu.ac.kr

Table of Contents

1. Table S1. Operating information of MALDI-TOF/TOF for analysis of Gd-DOTA-click-SF and elastase.

2. Figure S1. Low-resolution FAB mass spectrum of Cu-DOTA click SF.
3. Figure S2. The purity of the final product was checked using HPLC/PDA (A) and mass confirmation was performed with UPLC/TOF-MS (B).

4. Figure S3. Plots of relaxation rate as a function of gadolinium concentration (0.01, 0.02, 0.04, 0.08, 0.16, 0.32, and 0.64 mM Gd). The slopes correspond to the molar longitudinal relaxivity (r_1) of Dotarem (r_1 = 3.99 $mM^{-1}s^{-1}$) and Gd-DOTA-click-SF (r_1 = 7.04 $mM^{-1}s^{-1}$) obtained using the inversion recovery method.

5. Figure S4. MALDI-TOF/TOF mass calibration mix 3 in linear mode.

6. Figure S5. MALDI-TOF/TOF mass spectrum of Gd-DOTA-click-SF (reflector mode).

7. Figure S6. MALDI-TOF/TOF mass spectrum of Gd-DOTA-click-SF reaction with somatostatin (MW 1637.88) (reflector mode).

8. Figure S7. MALDI-TOF/TOF mass spectrum of Gd-DOTA-click-SF reaction with Oxytocin (MW 1007.19) (reflector mode).

9. Figure S8. MALDI-TOF/TOF mass spectrum of Gd-DOTA-click-SF reaction with ribonuclease A (MW 13683.30) (reflector mode).

10. Figure S9. Histopathologic analysis comparison of healthy control group and abdominal aortic aneurysm (AAA) group. Hematoxylin and Eosin staining (A), diameter (B), Verhoeff–Van Gieson (C), elastin content (D), trichrome (E), and collagen content (F). Healthy group (n = 3) and AAA group (n = 6). Welch t-test, **P < 0.01 and ****P < 0.0001. Scale bars: 100 μm (full aorta) and 50 μm (zoomed-in portion).

11. Figure S10. Correlation of ex vivo MRI and histopathology of ruptured rat AAA. (A) Picture of rat ruptured AAA. (B) Histopathological staining of hematoxylin and eosin for general morphology evaluation (B1), Verhoeff–Van Gieson for elastin (B2), and trichrome for collagen component identification (B3). (C) Ex vivo T1-weighted MR images of the same segment of rat ruptured AAA.

Instrument	MALDI-TOF/TOF™ 5800 system (AB SCIEX)
MS Parameters	
Operating Mode	MS Linear mode (Positive)
Mass Range (m/z)	5 ~ 50 kDa
Matrix (Conc. and Solution)	Sinapinic acid (SA) : 10 mg/mL (0.1% TFA/30% acetonitrile)
MS Calibration : mix3	Insulin (bovine) (charge +1, Average 5,734.59)
	Thioredoxin (E.coli) (charge +1, Average 11,674.48)
	Apomyoglobin (horse) (charge +1, Average 16,952.56)
	Apomyoglobin (horse) (charge +2, Average 8,476.78)
Sample preparation	matrix : sample = 29:1
Operating Mode	MS Reflector mode (Positive)
Mass Range (m/z)	800 ~ 4000 Da
Matrix (Conc. 및 Solution)	α-Cyano-4-hydroxycinnamic acid: 5 mg/mL (0.1% TFA/50% ACN)
MS Calibration mixture	Arg1-Bradykinin (904.468)
	Angiotensin I (1296.685)
	Glu1-Fibrinopeptide B (1570.677)
	ACTH (1-17) (2093.087)
	ACTH (18-39) (2465.199)
	ACTH (7-38) (3657.9294)
MS calibration	m/z 0.1
Sample preparation	matrix : sample = 1:1
Data processing	Baseline correction<br>Noise filter/smooth (Gaussian smooth 9 points)<br>Mass calibration

Table S1. Operating information of MALDI-TOF/TOF for analysis of Gd-DOTA-click-SF and elastase.

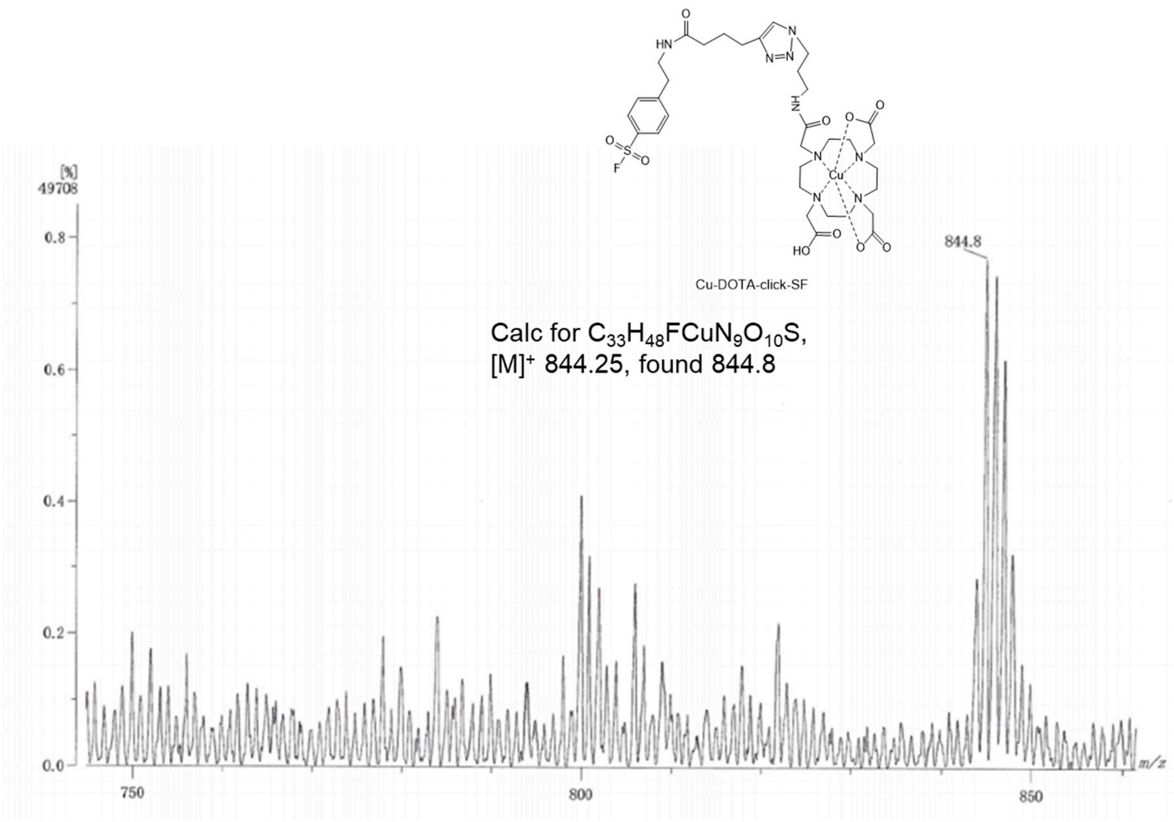

Figure S1. Low-resolution FAB mass spectrum of Cu-DOTA click SF.

Confirmation of the final product's purity and mass.

The purity of final product was checked using analytical high-performance liquid chromatography (HPLC) (Water Alliance 2695 (Waters, USA) photodiode array detector (PDA, Waters 2996), Avantor ® ACE ® C18 column (250 x 4.6 mm, 5 µm). Flow rate: 1 mL/min. Mobile phase used: Solvent A 0.1% trifluoroacetic acid (TFA) in water, solvent B 0.1% trifluoroacetic acid (TFA) in acetonitrile, (gradient condition 10% B in 2 min, 10-90% B in 13 min, 90% B in 5 min, 90-10% B in 5 min). According to the HPLC/PDA result, the purity of our product (Peak at 9.624 min, wavelength 228 nm) more than 97% (Figure S2A). The main peak was confirmed again by UPLC/TOF-MS (Acquity UPLC system, Synapt G2-Si HDMS (Waters, USA)). According to the result, two precursors SF-alkyne (mass 297.08) and Gd-azido-DOTA (mass 642.164) were not observed in the final product. The desired product was appeared as the major peak with mass was calculated for $C_{33}H_{47}FGdN_9O_{10}S$ $[M + H]^+$: 939.2476; found 939.248. The detail of method was described with Waters Acquity BEH C18 1.7 µm (2.1 x 100 mm). Column Temperature: 40 °C. Flow rate: 0.4 mL/min. Mobile phase used: Solvent A 0.1% formic acid (FA) in water, solvent B 0.1% formic acid (FA) in acetonitrile, (gradient condition 5% B in 1 min, 5-50% B in 7 min, 50-99% B in 0.2 min, 99% keep in 1.8 min, 99-5% B in 0.2 min, 5% B keep in 1.8 min). MS Mode: ESI (+), sensitivity mode, scan time: 0.15. Capillary (kV): 2.0 kV. Source Temperature: 110 °C. Devolvation temperature: 400 °C. Desolvation gas flow (L/Hr): 800. MS Scan range: 60-1400 Da.

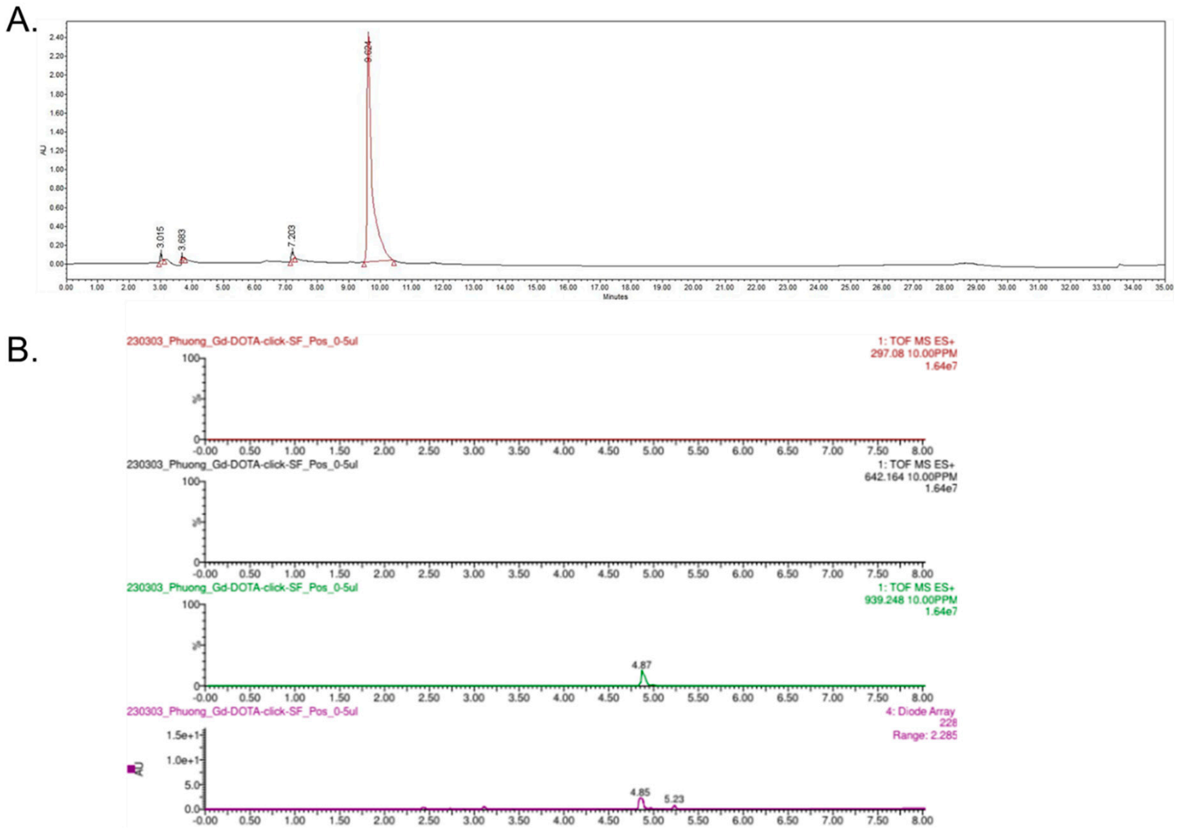

Figure S2. The purity of the final product was checked using HPLC/PDA (A) and mass confirmation was performed with UPLC/TOF-MS (B)

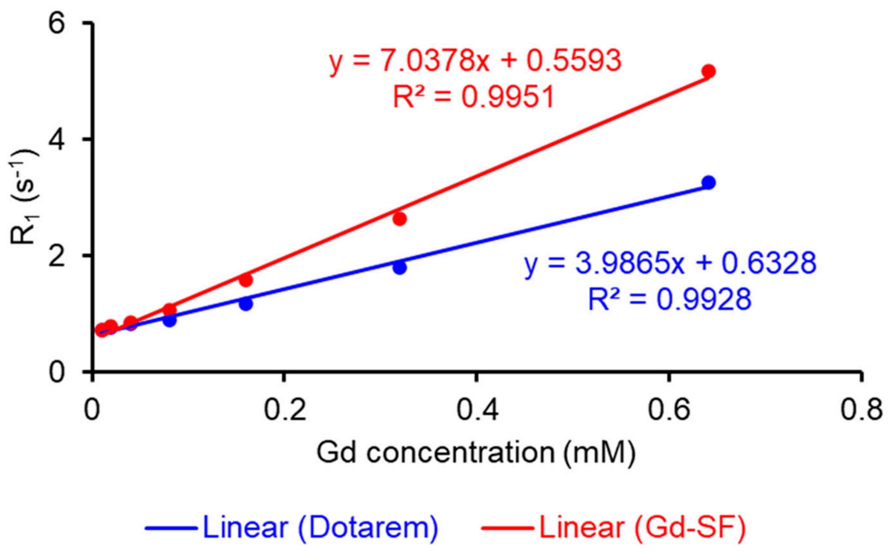

Figure S3. Plots of relaxation rate as a function of gadolinium concentration (0.01, 0.02, 0.04, 0.08, 0.16, 0.32, and 0.64 mM Gd). The slopes correspond to the molar longitudinal relaxivity (r_1) of Dotarem (r_1 = 3.99 $mM^{-1}s^{-1}$) and Gd-DOTA-click-SF (r_1 = 7.04 $mM^{-1}s^{-1}$) obtained using the inversion recovery method.

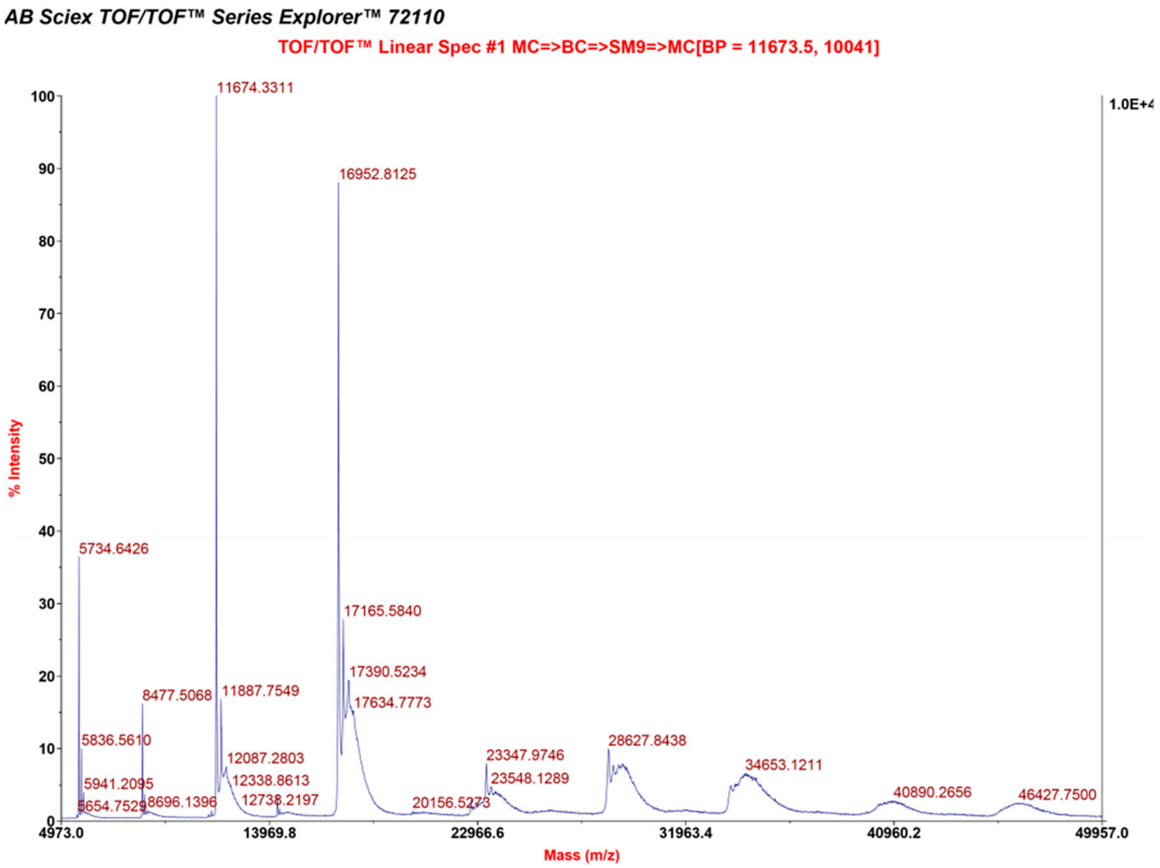

Figure S4. MALDI-TOF/TOF mass calibration mix 3 in linear mode

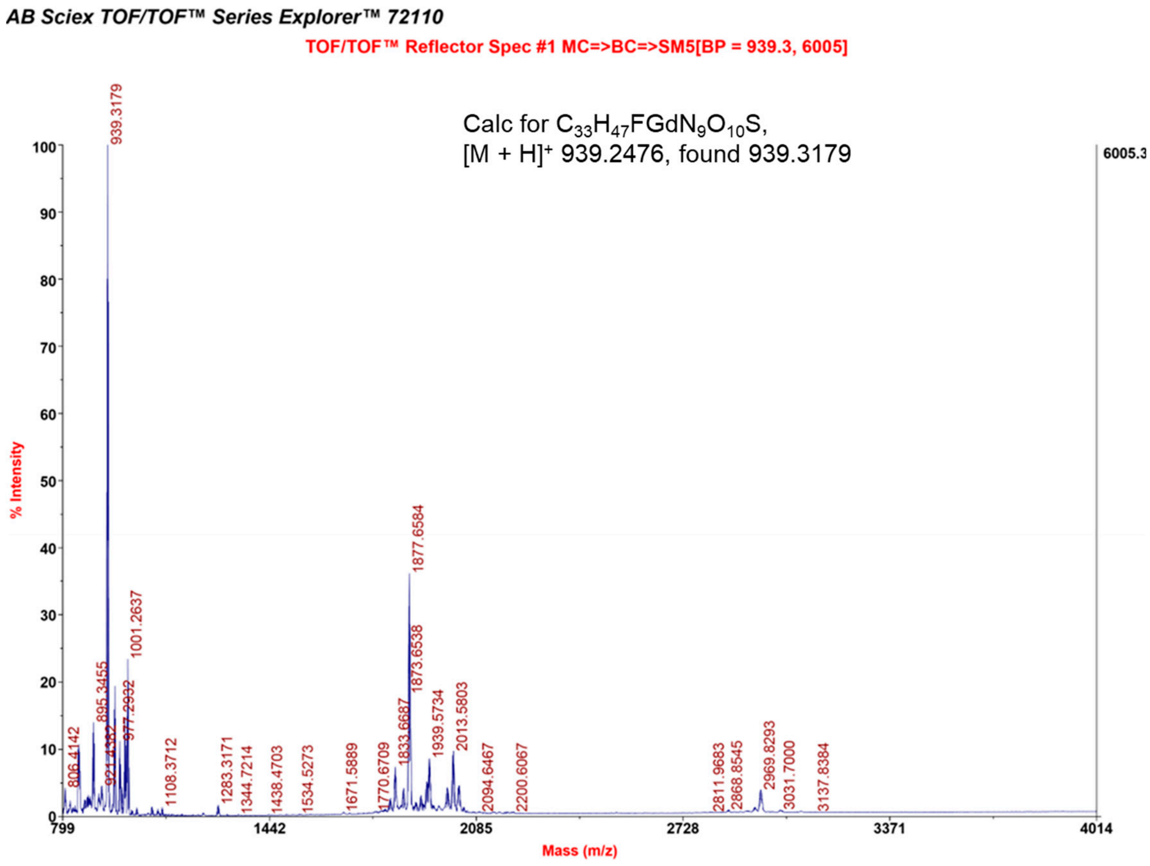

Figure S5. MALDI-TOF/TOF mass spectrum of Gd-DOTA-click-SF (reflector mode)

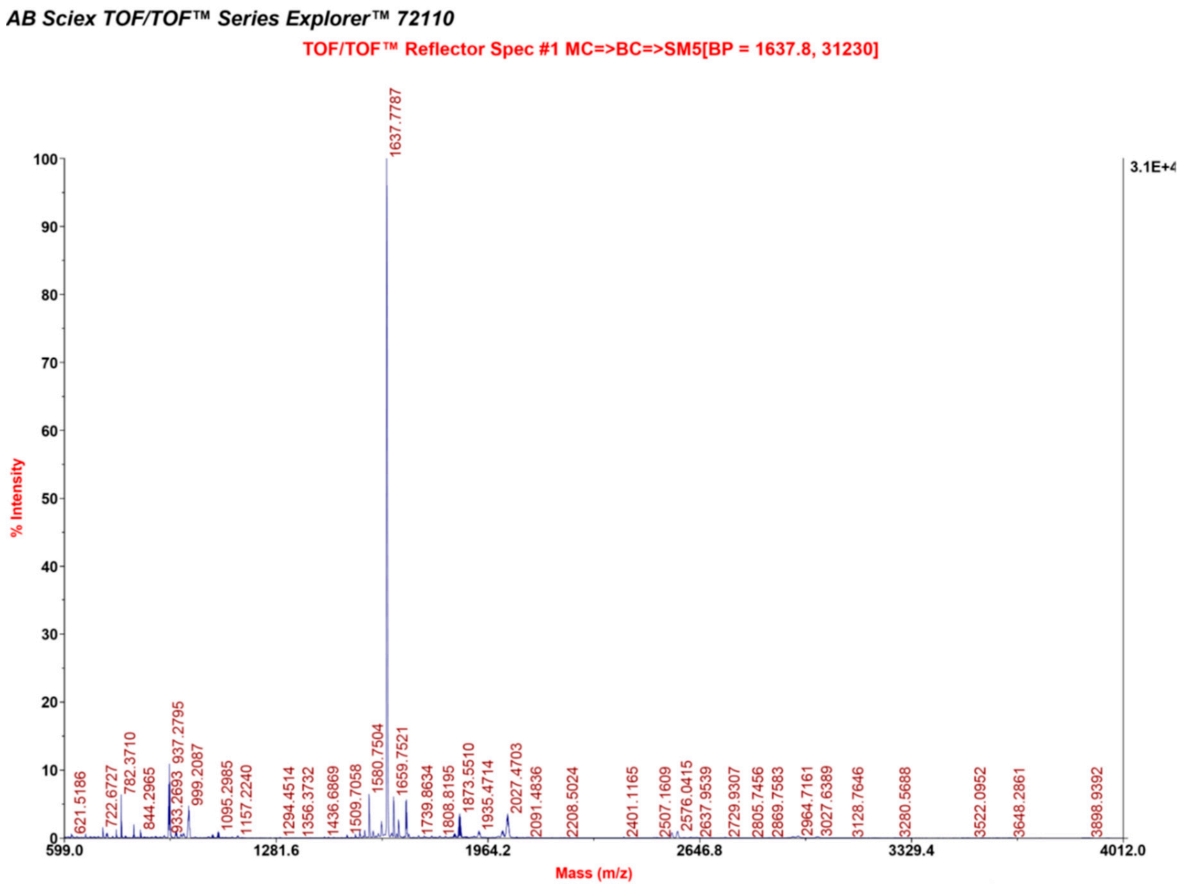

Figure S6. MALDI-TOF/TOF mass spectrum of Gd-DOTA-click-SF reaction with somatostatin (MW 1637.88) (reflector mode)

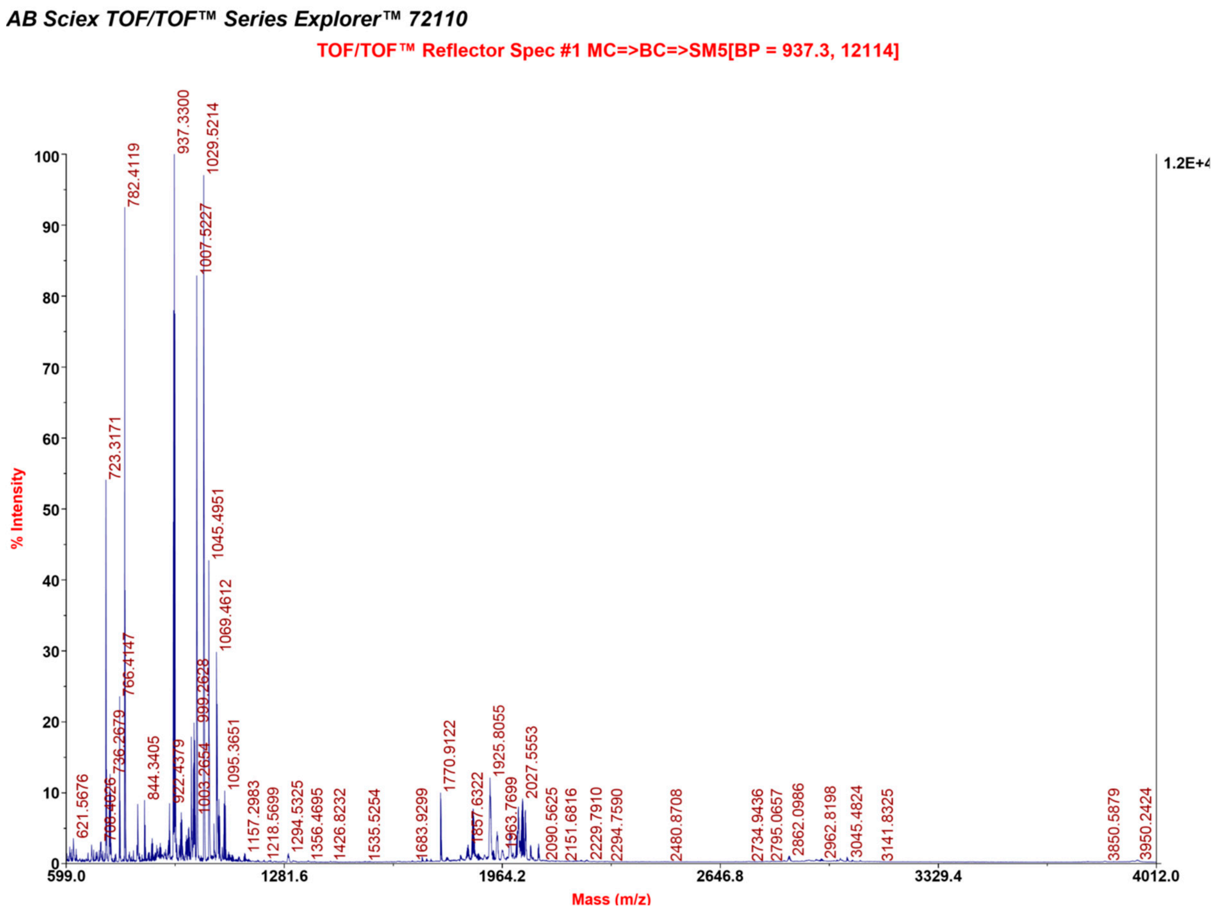

Figure S7. MALDI-TOF/TOF mass spectrum of Gd-DOTA-click-SF reaction with Oxytocin (MW 1007.19) (reflector mode)

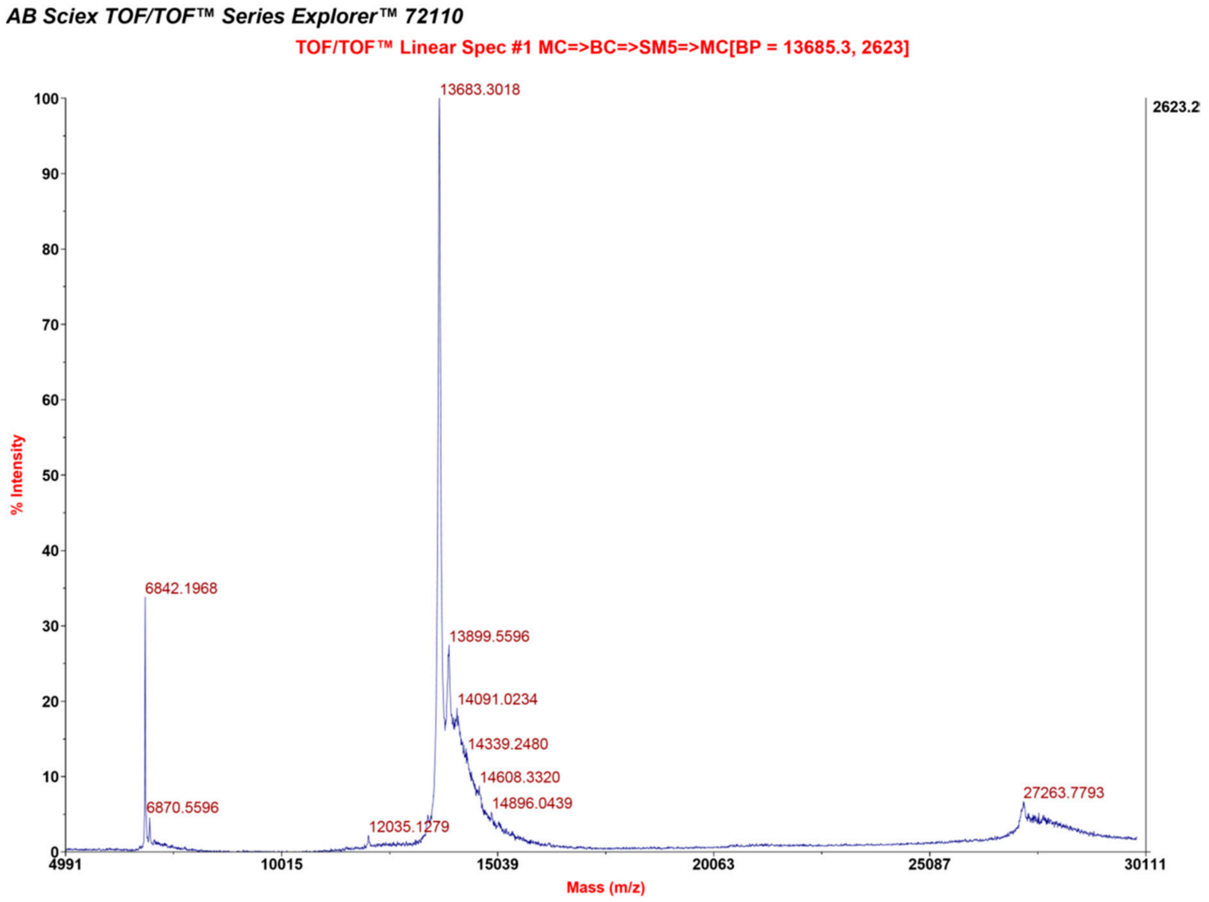

Figure S8. MALDI-TOF/TOF mass spectrum of Gd-DOTA-click-SF reaction with ribonuclease A (MW 13683.30) (reflector mode)

Histopathologic Analysis

In order to prove the successful development of AAA models, the histopathology of aorta specimens were performed by using staining kits such as hematoxylin and eosin for general morphology, Verhoeff–Van Gieson for elastin and trichrome for collagen examination.

According to the Figure S9, the diameter of AAA groups significant enlarger than that of healthy groups. Moreover, AAA groups showed significant degradation of elastin content and compensatory collagen overproduction which proved the AAA model development.

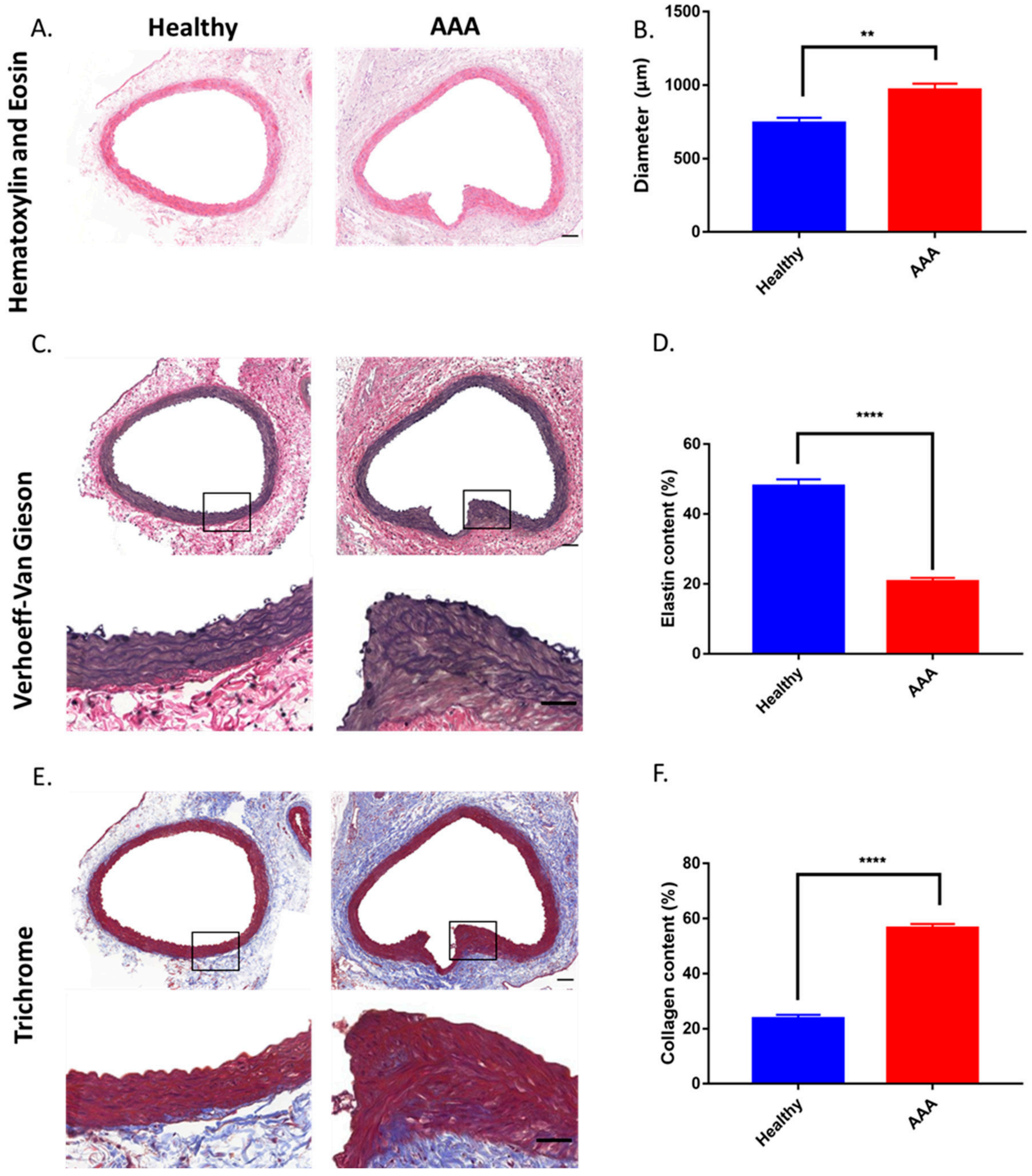

Figure S9. Histopathologic analysis comparison of healthy control group and abdominal aortic aneurysm (AAA) group. Hematoxylin and eosin staining (A), diameter (B), Verhoeff–Van Gieson (C), elastin content (D), trichrome (E), and collagen content (F). Healthy group (n = 3) and AAA group (n = 6). Welch t-test, **$P < 0.01$ and ****$P < 0.0001$. Scale bars: 100 µm (full aorta) and 50 µm (zoomed-in portion).

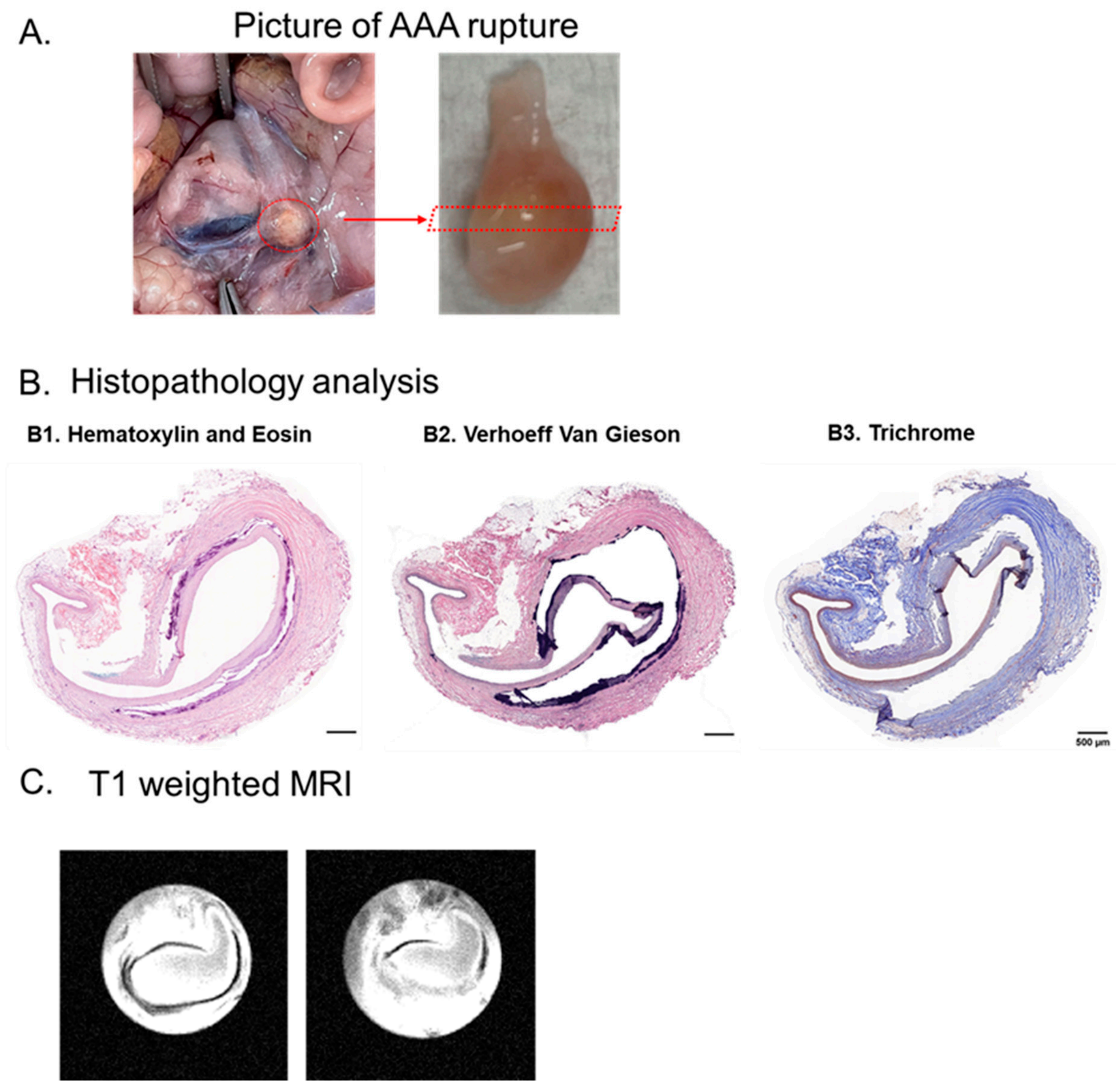

Figure S10. Correlation of ex vivo MRI with histopathology of ruptured rat AAA. (A) Picture of rat ruptured AAA. (B) Histopathologic staining with hematoxylin and eosin for general morphology evaluation (B1), Verhoeff–Van Gieson for elastin (B2), and trichrome for collagen component identification (B3). (C) Ex vivo T1-weighted MR images of the same segment of rat ruptured AAA.

Figure S10 illustrates an example of an MRI-derived morphometric measurement of the aneurysm. The ex vivo MRI of rat ruptured AAA was

correlated with histopathology stains. In detail, MR images were obtained for the lumen and remodeled vessel walls of the AAA in addition to the hematoxylin and eosin, elastin, and trichrome staining. The lumen appeared in dark by MRI and coregistered trichrome and elastin-stained section in bright T1-weighted imaging.

Reference

1. Gregory HT, Alan RO, Roberta EB, Karpagam A, Heather WK, Rosanna CM, Robert NW, Peter JG and Beat MJ. In Vivo Serial Assessment of Aortic Aneurysm Formation in Apolipoprotein E–Deficient Mice via MRI. *Circulation: Cardiovascular Imaging.* **2008**, 1:220–226. https://doi.org/10.1161/CIRCIMAGING.108.787358
